# Prognostic Value of Oral Epstein–Barr Virus DNA Load in Locoregionally Advanced Nasopharyngeal Carcinoma

**DOI:** 10.3389/fmolb.2021.757644

**Published:** 2022-01-13

**Authors:** Yong-Qiao He, Ting Zhou, Da-Wei Yang, Yi-Jing Jia, Lei-Lei Yuan, Wen-Li Zhang, Tong-Min Wang, Ying Liao, Wen-Qiong Xue, Jiang-Bo Zhang, Xiao-Hui Zheng, Xi-Zhao Li, Pei-Fen Zhang, Shao-Dan Zhang, Ye-Zhu Hu, Fang Wang, William C. Cho, Jun Ma, Ying Sun, Wei-Hua Jia

**Affiliations:** ^1^ State Key Laboratory of Oncology in South China, Collaborative Innovation Center for Cancer Medicine, Guangdong Key Laboratory of Nasopharyngeal Carcinoma Diagnosis and Therapy, Sun Yat-sen University Cancer Center, Guangzhou, China; ^2^ Biobank of Sun Yat-sen University Cancer Center, Guangzhou, China; ^3^ School of Public Health, Sun Yat-sen University, Guangzhou, China; ^4^ Department of Clinical Oncology, Queen Elizabeth Hospital, Hong Kong SAR, China

**Keywords:** Epstein–Barr virus, oral EBV load, nasopharyngeal carcinoma, survival, locoregionally advanced

## Abstract

**Background:** Plasma Epstein–Barr virus (EBV) DNA load has been widely used for nasopharyngeal carcinoma (NPC) prognostic risk stratification. However, oral EBV DNA load, a non-invasive biomarker that reflects the EBV lytic replication activity, has not been evaluated for its prognostic value in NPC yet.

**Methods:** A total number of 1,194 locoregionally advanced NPC (LA-NPC) patients from south China were included from a prospective observational cohort (GARTC) with a median follow-up of 107.3 months. Pretreatment or mid-treatment mouthwashes were collected for EBV DNA detection by quantitative polymerase chain reaction (qPCR). The difference of pre- and mid-treatment oral EBV DNA load was tested by the Wilcoxon signed-rank test. The associations of oral EBV DNA load with overall survival (OS), progression-free survival (PFS), distant metastasis–free survival (DMFS), and locoregional relapse-free survival (LRFS) were assessed using the log-rank test and multivariate Cox regression.

**Results:** The high level of the oral EBV DNA load (>2,100 copies/mL) was independently associated with worse OS (*HR* = 1.45, 95% *CI*: 1.20–1.74, *p* < 0.001), PFS (*HR* = 1.38, 95% *CI*: 1.16–1.65, *p* < 0.001), DMFS (*HR* = 1.66, 95% *CI*: 1.25–2.21, *p* = 0.001), and LRFS (*HR* = 1.43, 95% *CI*: 1.05–1.96, *p* = 0.023). Similar and robust associations between oral EBV DNA load and prognosis were observed for patients in both the pretreatment and mid-treatment stages. The detection rate (71.7 vs. 48.6%, *p* < 0.001) and the median load of oral EBV DNA (13,368 vs. 382 copies/mL, *p* < 0.001) for patients in the pretreatment stage were significantly higher than those in the mid-treatment stage. The combination of the oral EBV DNA load and TNM staging provided a more precise risk stratification for the LA-NPC patients.

**Conclusion:** Oral EBV DNA load was an alternative non-invasive predictor of prognosis and may facilitate risk stratification for the LA-NPC patients.

## Introduction

Nasopharyngeal carcinoma (NPC) is characterized by its distinguished geographical distribution, with more than 70% of all worldwide cases located in south China and southeast Asia (Tang et al., 2016a; Bray et al., 2018). Early-stage NPC is relatively asymptomatic; thus, nearly 80% of patients present locally advanced disease with poor prognosis (Pan et al., 2016; Lee et al., 2017). Given the advanced disease stage and the peak occurrence at a relatively young age of 40–65 y, NPC contributes prominently to the cancer burden with substantial economic and societal impacts in endemic areas. Since studies have suggested that additional treatment such as induction chemotherapy (IC) could improve the prognosis of patients with advanced NPC (Frikha et al., 2018; Hong et al., 2018; Li et al., 2019), it is crucial to identify patients with poor prognoses so that timely guided therapeutic strategies could be implemented. The clinical stage at diagnosis is one of the most important indicators for NPC prognosis. However, due to tumor heterogeneity, traditional anatomy–based TNM staging is inadequate for accurately predicting the prognosis or therapeutic benefits of patients with locoregionally advanced NPC (Leung et al., 2006). This highlights the importance of identifying feasible biomarkers to help risk stratification and risk-adapted therapeutic strategy modification for the long-term follow-up of NPC survivors.

Various blood biomarkers, such as Epstein–Barr virus (EBV) DNA, EBV antibodies, and microRNAs, have been explored as prognostic indicators for NPC (Liu et al., 2014; Tang et al., 2015; Yao et al., 2018; Cai et al., 2019). Plasma EBV DNA, which is mainly released from the tumor cells, is a robust biomarker for prognosis prediction and disease surveillance of NPC (Lo et al., 2000; Lin et al., 2004; Tang et al., 2016b; Sun et al., 2019). Regarding NPC being located at the nasopharynx of the head, apart from the circulating peripheral blood system, saliva is another important liquid biopsy for detecting the present of EBV. We know that EBV infection in the oral epithelial tissue tends to lead to productive lytic replication (Young et al., 2016); hence, the oral EBV DNA load can reflect the host’s EBV lytic replication activity and may be potentially correlated with the development of EBV-associated diseases. However, the relationship between oral EBV DNA and NPC prognosis was largely unknown.

Although plasma EBV DNA has been used for NPC prognosis, the sampling method by venipuncture is an invasive procedure that might cause complications, for example, potential infection and development of bruises where the procedure took place, which is harmful to the cancer patients. On the contrary, saliva or mouthwash collection is non-invasive and can be easily conducted without a professional phlebotomist and operations. This supports the rationale to investigate whether oral EBV DNA might be an alternative method for NPC prognosis prediction. To the best of our knowledge, this is the first study focusing on the prognostic value of oral EBV DNA in NPC patients. Therefore, based on our present prospective cohort comprising relatively large sample size and long follow-up time, we aimed to explore whether the baseline oral EBV DNA load was able to independently predict the prognosis of LA-NPC patients to guide risk stratification.

## Materials and Methods

### Study Population

**FIGURE 1:**
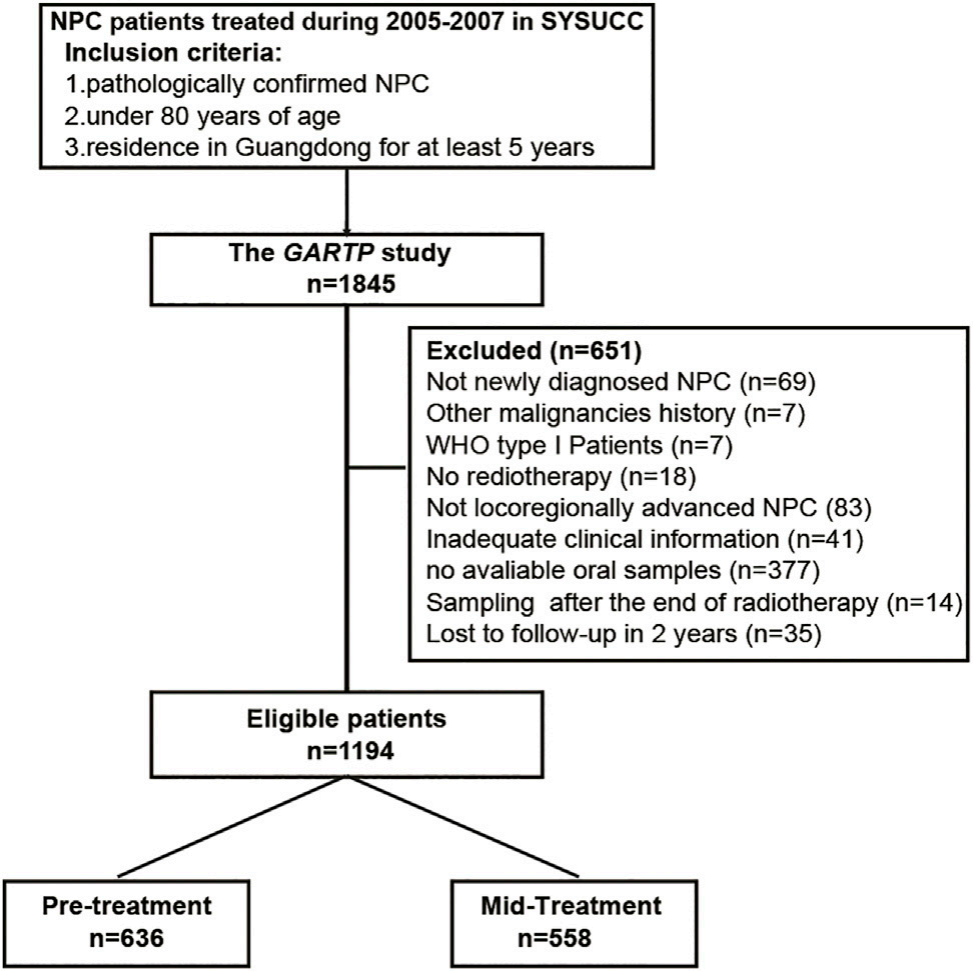
Flowchart of patient inclusion and exclusion. NPC = nasopharyngeal carcinoma.

The GARTP study (ChiCTRROC-17012658) is a prospective observational clinical trial that aims to identify the risk factors contributing to disease prognosis and radiation-induced complications in NPC patients (Wang et al., 2019). The primary cohort recruited 1,845 NPC patients under 80 y of age who were residing in Guangdong for at least 5 y from Sun Yat-sen University Cancer Center (SYSUCC, Guangzhou, China) during October 2005 and October 2007 (Xu et al., 2012; Xue et al., 2018). Our study included 1,194 eligible patients from the GARTP cohort according to the following criteria: 1) histology-confirmed NPC of WHO type II or III; 2) locoregionally advanced NPC (TxN2-3M0 or T2-4N0-3M0 according to the 6th AJCC/UICC TNM staging systems); 3) no other EBV-associated diseases, such as T-cell lymphoma, natural killer cell leukemia/lymphoma, Hodgkin’s disease, pyothorax-associated B-cell lymphoma, gastric carcinoma, and HIV infection; 4) received radiotherapy with or without chemotherapy; 5) available mouthwashes collected before the completion of radiotherapy; 6) available necessary clinical information; and 7) no loss to follow-up within 2 y. The flowchart is shown in Figure 1. This study was approved by the Ethics Committee of the SYSUCC, and written informed consent was all obtained at the patient enrollment. Study data were deposited at the Research Data Deposit platform (RDDA2020001573).

### Oral EBV DNA Assay

The oral EBV DNA load was measured in the collected mouthwashes before the commencement of any therapy (pretreatment), or between the start of treatment and the end of radiotherapy/chemoradiotherapy (mid-treatment). All mouthwash samples were collected and processed by standard procedures as reported in our previous studies (Xue et al., 2018; He et al., 2019). Briefly, 30 min before sampling, the patients were advised not to eat or drink anything. Then, each patient was instructed to rinse their mouth with 10 mL of physiological saline (0.9% NaCl) for 30 s. EBV DNA was isolated from mouthwash using a phenol–chloroform procedure and measured by quantitative polymerase chain reaction (qPCR), with primers and probe targeting a 76-bp segment in the BamHI-W region of the EBV genome, which were consistent with those commonly used for plasma EBV DNA quantification (Lo et al., 1999). Plasmid DNA containing the target region in serial dilution was used as a standard for absolute quantification. The oral EBV DNA load was expressed as the copies of EBV DNA per mL of mouthwash (Pow et al., 2011; He et al., 2019). More detailed methods are described in Supplementary Method. All testing samples and quantification standard samples were tested in duplicate, and the coefficient of variance (CV) and the intraclass correlation coefficient (ICC) were estimated to evaluate the reproducibility of the EBV DNA test. The median CVs for retesting samples and quantification standards were 0.34 and 0.52%, respectively. The ICCs for retesting samples and quantification standards were 88.84 and 99.63%, respectively.

### Clinical Assessment and Treatment

Before treatment, patients underwent a complete baseline evaluation, including physical examination, fiberoptic nasopharyngoscopy, magnetic resonance imaging (MRI) of the nasopharynx and neck, biochemistry and hematology profiles, chest radiography or tomography, abdominal sonography, and bone scintigraphy. The treatment administration was conducted following the routine practice of SYSUCC. All patients underwent radiotherapy, and the prescribed radiation doses were 68–74 Gy for primary tumor and 50–66 Gy for regional lymph nodes. Since the patients were enrolled before 2007 when the role of chemotherapy, such as concurrent chemoradiotherapy (CCRT) and induction chemotherapy (IC), has not been well established, there was no consensus on chemotherapy, and it mainly depended on the experience and the decisions of clinicians.

### Outcome and Follow-Up

After completing treatment, the patients attended regular follow-up every 3 months during the first 2 y, every 6 months during 3–5 y, and annually thereafter. Locoregional/distant failure was confirmed by nasopharyngeal biopsy or two radiographic diagnoses (CT\MRI\ECT) not more than 3 months apart. They were also followed up through telephone interviews and death registration at the public security bureau if their recent medical records were not recorded. The last follow-up date was September 2019. The primary endpoint was overall survival (OS, time for the NPC diagnosis to death from any cause or censored at the date of the last follow-up). The secondary endpoints included progression-free survival (PFS, time from NPC diagnosis to the first event or death from any cause or censored at the date of the last follow-up), locoregional relapse-free survival (LRFS, time from NPC diagnosis to the first locoregional recurrence or censored at the date of the last follow-up), and distant metastasis–free survival (DMFS, time from NPC diagnosis to first distant failure or censored at the date of the last follow-up).

### Statistical Analyses

All patients were first categorized into either the low EBV (≤2,100 copies/mL) or high (>2,100 copies/mL) EBV groups, according to the optimum cutoff value determined by the receiver operating characteristic (ROC) curve analysis for OS in all subjects, and the cutoff value was set to 2,100 copies/mL rather than the calculated 2,086 copies/mL for its potential acceptance and clinical application. According to the treatment stage at which the mouthwash samples were collected, patients were further grouped into pre- and mid-treatment subsets. Since the distribution of data on the oral EBV load was highly skewed, the oral EBV DNA load was log10 transformed and compared between the pretreatment subset and mid-treatment subset by the Wilcoxon rank-sum test. The univariate and multivariate logistic regressions were performed to assess the associations between clinical characteristics and EBV DNA levels. Survival curves were depicted using the Kaplan–Meier method and compared using the log-rank test. Multivariate Cox regression analyses include age, sex, smoking status, T stage, N stage, IC, CCRT, radiation technology, and oral EBV DNA level. To describe the dose–effect relationship between the oral EBV DNA load and NPC survivals, EBV DNA copy numbers were log10 transformed in the COX regressions as a continuous variable. Statistical significance was defined as two-sided *p* < 0.05. Analyses were performed using R software, version 4.0.2 (http://www.r-project.org).

## Results

### Patient Characteristics

The total study population comprised 1,194 patients with locoregionally advanced NPC. There were 864 (72.4%) males and 330 (27.6%) females with a median age of 45 y. Among them, 375 patients received RT alone; 254 received platinum-based CCRT; 349 received 1–2 cycles of TP or PF, IC, and RT; and 216 received IC followed by CCRT. The median follow-up time was 107.3 (range 3.5–164.6) months. The 5-y survival rates for OS, PFS, DMFS, and LRFS were 77.5, 69.7, 83.1, and 88.0%, respectively. According to the treatment stage at which the mouthwash samples were collected, 636 patients were grouped into the pretreatment subset and 558 patients were in the mid-treatment subset (Table 1).

**TABLE 1:** Clinical characteristics of 1,194 locoregional advanced NPC patients.

Variables	Total, No (%)
Age [mean (SD)]	45.88 (10.90)
Sex	
Female	330 (27.6)
Male	864 (72.4)
Smoking status	
No	563 (47.2)
Yes	631 (52.8)
Tumor stage	
T1	40 (3.4)
T2	292 (24.5)
T3	575 (48.2)
T4	287 (24.0)
N stage	
N0	276 (23.1)
N1	495 (41.5)
N2	353 (29.6)
N3	70 (5.9)
Overall stage	
II	208 (17.4)
III	645 (54.0)
IV	341 (28.6)
IC	
No	629 (52.7)
Yes	565 (47.3)
CCRT	
No	724 (60.6)
Yes	470 (39.4)
RT technology	
2DRT	947 (79.3)
3DCRT	29 (2.4)
IMRT	153 (12.8)
Not clear	65 (5.4)
Sampling stage^a^	
Pretreatment	636 (53.3)
Mid-treatment	558 (46.7)

a Pretreatment, before any treatment; mid-treatment, between the start of treatment and the end of radiotherapy/chemoradiotherapy.

NPC, nasopharyngeal carcinoma; IC, induction chemotherapy; CCRT, concurrent chemoradiotherapy; RT, radiotherapy, 2DRT, two-dimensional radiotherapy; 3DCRT, three-dimensional conformal radiotherapy; IMRT, intensity-modulated radiotherapy.

### Correlation Between the Oral EBV DNA Level and Clinical Characteristics

EBV DNA could be detected in 456 (71.7%) patients in the pretreatment subgroup and 271 (48.6%) patients in the mid-treatment subgroup (*p* < 0.001). And the median oral EBV DNA load of mid-treatment samples was significantly lower than that of pretreatment ones (382 vs. 13,368 copies/mL, Wilcoxon *p* < 0.001, Supplementary Figure 1A). Univariate and multivariate logistic analyses of pretreatment, mid-treatment, and overall patients showed that patients with older age (≥45 y), male gender, and advanced T stage (T3–4) tended to have a higher copy number of oral EBV DNA, albeit with some associations not reaching the significant threshold. In contrast, patients with the advanced N stage were more likely to present a lower level of the oral EBV DNA load (Table 2, Supplementary Table 1).

**TABLE 2:** Multivariate associations of clinical characteristics with the oral Epstein–Barr virus DNA load at different treatment time points in nasopharyngeal carcinoma patients.

Variables	Pretreatment^a^, *n* = 636		Mid-treatment^a^, *n* = 558		Overall^a^, *n* = 1,194	
	OR (95% *CI*)^b^	*P* ^c^	OR (95% *CI*)^b^	*p* ^c^	OR (95% *CI*)^b^	*P* ^c^
Age (≥45 vs.<45)	1.37 (0.98–1.92)	0.062	1.51 (1.06–2.14)	**0.021**	1.44 (1.14–1.83)	**0.002**
Sex (male vs. female)	2.04 (1.27–3.31)	**0.004**	1.63 (0.99–2.71)	0.056	1.75 (1.25–2.45)	**0.001**
Smoking status (yes vs. no)	0.84 (0.54–1.31)	0.456	1.10 (0.70–1.71)	0.686	0.97 (0.72–1.32)	0.870
Tumor stage (T3–4 vs. T1–2)	1.71 (1.20–2.44)	**0.003**	1.62 (1.08–2.45)	**0.022**	1.53 (1.18–1.98)	**0.001**
N stage (N2–3 vs. N0–1)	0.65 (0.46–0.93)	**0.018**	0.68 (0.47–0.97)	**0.032**	0.63 (0.49,0.81)	**<0.001**

a Pretreatment, before any treatment; mid-treatment, between the start of treatment and the end of radiotherapy/chemoradiotherapy; overall, all patients included.

b Multivariate logistic analysis with covariates of age, sex, smoking status, T stage, and N stage.

c P values less than 0.05 are shown in bold.

### Prognostic Role of Oral EBV DNA in LA-NPC Patients

**FIGURE 2:**
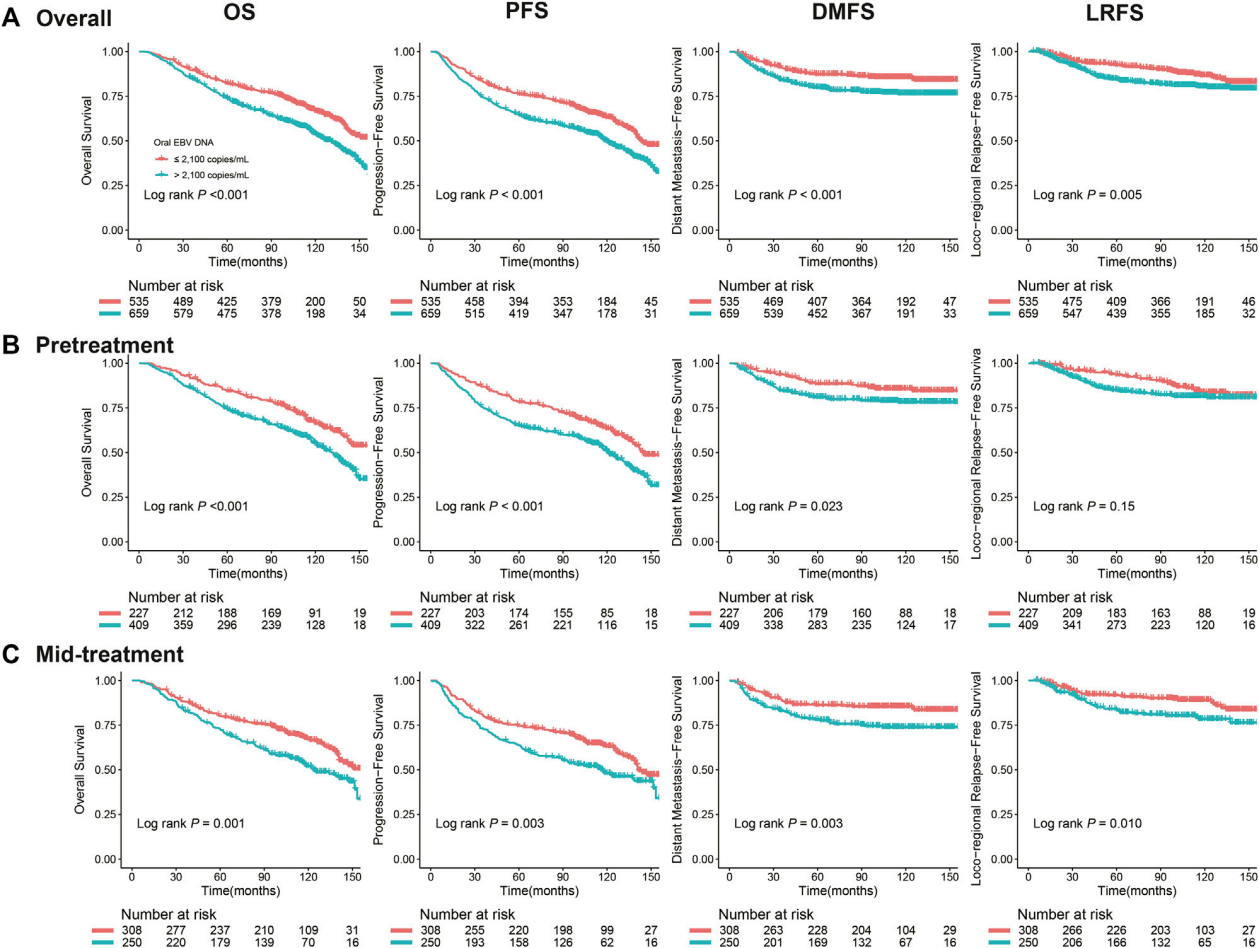
Kaplan–Meier curves for NPC survival segregated by the oral EBV DNA level at different treatment time points: **(A)** overall, **(B)** pretreatment, before any treatment, **(C)** mid-treatment, between the start of treatment and the end of radiotherapy/chemoradiotherapy. Survivals included overall survival (OS), progression-free survival (PFS), distant metastasis–free survival (DMFS), and locoregional relapse-free survival (LRFS).

We observed that patients with a high level of the oral EBV DNA load (>2,100 copies/mL) had inferior OS (5-y rate, 74.0 vs. 82.0%, log-rank *p* < 0.001), PFS (64.6 vs. 76.1%, log-rank *p* < 0.001), DMFS (79.8 vs. 87.1%, log-rank *p* < 0.001), and LRFS (84.4 vs. 92.4%, log-rank *p* = 0.005) than patients with a low oral EBV DNA (≤2,100 copies/mL) (Figure 2A). Further stratified according to the treatment stage, similar trends were also observed in both pretreatment and mid-treatment subsets. In the pretreatment subset, high-EBV patient had inferior 5-y OS (74.6 vs. 84.5%, log-rank *p* < 0.001), PFS (65.2 vs. 78.3%, log-rank *p* < 0.001), and DMFS (80.9 vs. 88.4%, log-rank *p* = 0.023) than patients with low oral EBV, while the 5-y LRFS rate was comparable between patients with high oral EBV and those with low oral EBV (85.0 vs. 93.4%, log-rank *p* = 0.150) (Figure 2B). In the mid-treatment subset, the 5-y OS rate (73.0 vs. 80.3%, log-rank *p* = 0.001), PFS rate (63.6 vs. 74.5%; log-rank *p* = 0.003), DMFS rate (78.0 vs. 86.2%, log-rank *p* = 0.003), and LRFS rate (83.5 vs. 91.6%, log-rank *p* = 0.010) for high-EBV patients were significantly lower than the corresponding rates for low-EBV patients (Figure 2C).

Multivariate analyses suggested that the high level of the oral EBV DNA load was independently associated with worse OS (*HR* = 1.45, 95% *CI*: 1.20–1.74, *p* < 0.001), PFS (*HR* = 1.38, 95% *CI*: 1.16–1.65, *p* < 0.001), DMFS (*HR* = 1.66, 95% *CI*: 1.25–2.21, *p* = 0.001), and LRFS (*HR* = 1.43, 95% *CI*: 1.05–1.96, *p* = 0.023). The pretreatment EBV DNA copy numbers were log10 transformed as a continuous variable, and significant dose–effect relationships of the oral EBV DNA load with all four outcomes were observed (*p* = 0.001∼0.012). Similar associations between the oral EBV DNA load and NPC survivals were observed for patients in both the pretreatment and mid-treatment subgroups, except that no significant association was observed between pretreatment oral EBV DNA and LRFS (Table 3).

**TABLE 3:** Multivariate Cox regression analysis of the oral EBV DNA load with NPC survivals.

Oral EBV DNA load^a^	OS		PFS		DMFS		LRFS	
	*HR* (95% *CI*)^b^	*p* ^c^	*HR* (95% *CI*)^b^	*p* ^c^	*HR* (95% *CI*)^b^	*p* ^c^	*HR* (95% *CI*)^b^	*p* ^c^
Overall^d^								
>2,100 vs. ≤2,100 copy/mL	1.45 (1.20–1.74)	**<0.001**	1.38 (1.16–1.65)	**<0.001**	1.66 (1.25–2.21)	**0.001**	1.43 (1.05–1.96)	**0.023**
Log10 transformed EBV load	1.07 (1.03–1.11)	**0.001**	1.06 (1.02–1.10)	**0.001**	1.10 (1.03–1.16)	**0.002**	1.09 (1.02–1.16)	**0.012**
Pretreatment^d^								
>2,100 vs. ≤2,100 copy/mL	1.51 (1.16–1.98)	**0.002**	1.43 (1.11–1.84)	**0.006**	1.60 (1.05–2.45)	**0.030**	1.22 (0.79–1.89)	0.377
Log10 transformed EBV load	1.10 (1.03–1.16)	**0.002**	1.08 (1.02–1.14)	**0.007**	1.09 (0.99–1.19)	0.064	1.06 (0.96–1.16)	0.255
Mid-treatment^d^								
>2,100 vs. ≤2,100 copy/mL	1.35 (1.03–1.76)	**0.030**	1.31 (1.02–1.70)	**0.037**	1.77 (1.18–2.64)	**0.006**	1.66 (1.05–2.62)	**0.030**
Log10 transformed EBV load	1.04 (0.99–1.10)	0.136	1.04 (0.99–1.09)	0.114	1.10 (1.02–1.19)	**0.015**	1.11 (1.01–1.21)	**0.025**

a EBV DNA load in mouthwashes.

b Adjusted by age (≥45 vs. <45), sex, T stage, N stage, smoking status (yes or no), IC (yes or no), CCRT (yes or no), and radiotherapy technology.

c P values less than 0.05 are shown in bold.

d Overall, all patients included; pretreatment, before any treatment; mid-treatment, between the start of treatment and the end of radiotherapy/chemoradiotherapy.

EBV, Epstein–Barr virus; NPC, nasopharyngeal carcinoma; OS, overall survival; PFS, progression-free survival; DMFS, distant metastasis–free survival; LRFS, locoregional relapse-free survival; IC, induction chemotherapy; CCRT, concurrent chemoradiotherapy.

We have tried to divide the whole population into three subgroups instead of two, namely, pretreatment (sampling before any treatment), post-IC (sampling between the start of IC and the start of radiotherapy/chemoradiotherapy), and mid-RT (sampling during radiotherapy/chemoradiotherapy). We found no significant difference in the oral EBV DNA load between the post-IC and mid-RT subgroups (Wilcoxon *p* = 0.340, Supplementary Figure 1B); thus, we combined the two groups to form the mid-treatment group instead. To test the robustness of our results, we performed further analyses by setting different cutoffs for the oral EBV DNA load. These results all showed similar trends that high copy number of oral EBV DNA was an independent predictor of poor prognosis for LA-NPC patients (Supplementary Table S2). And we have performed further analyses by breaking the oral EBV DNA load into three and four levels, and the results consistently supported the dose–response relationships between the oral EBV DNA load and NPC survivals (Supplementary Table S3).

### Patient Outcomes in Subgroups Defined by Oral EBV DNA Combined With the TNM Stage

**FIGURE 3:**
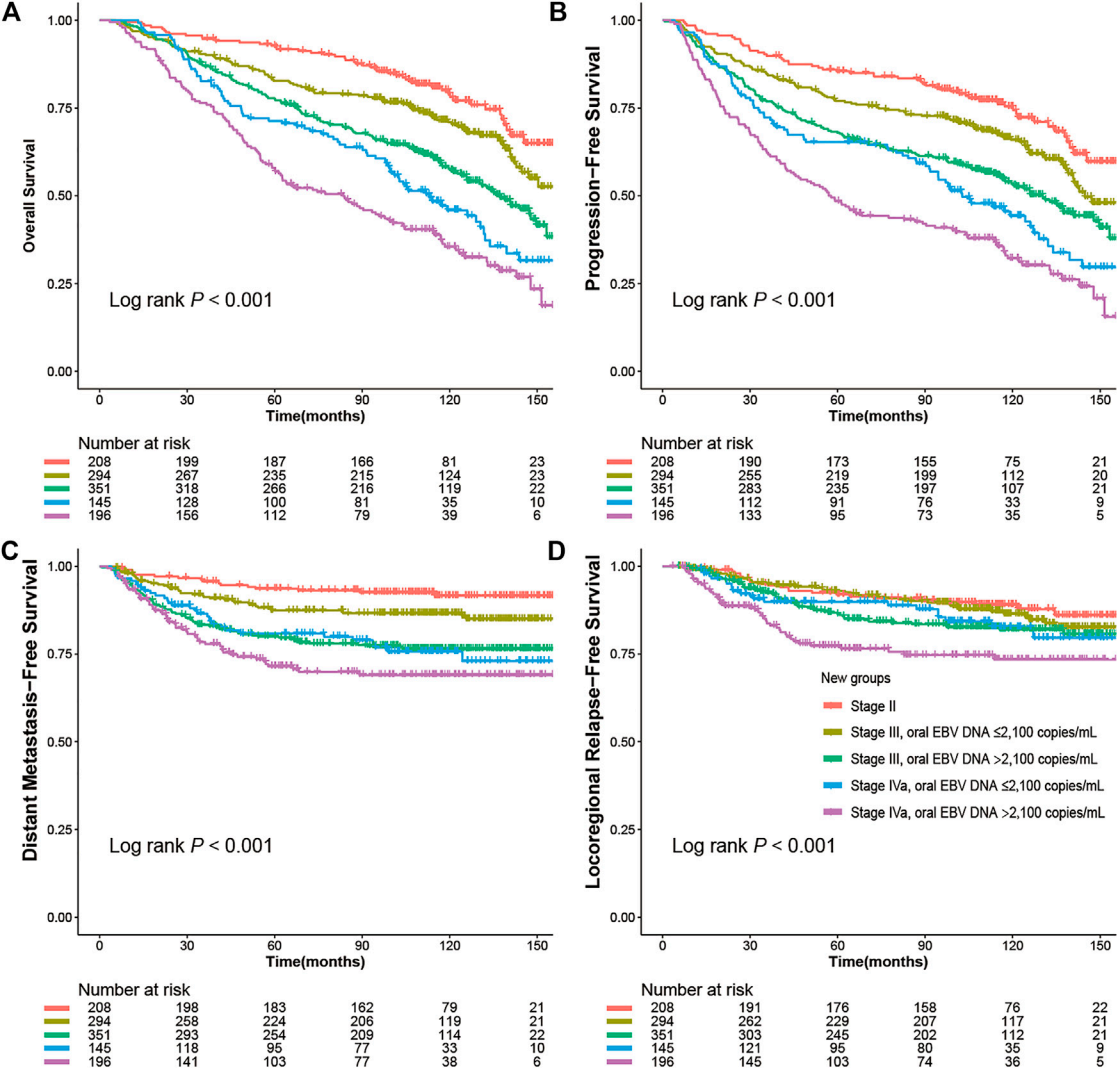
Kaplan–Meier survival curves for NPC survivals segregated by the oral EBV DNA level and TNM staging in all 1,194 NPC patients: **(A)** overall survival (OS), **(B)** progression-free survival (PFS), **(C)** distant metastasis–free survival (DMFS), **(D)** locoregional relapse-free survival (LRFS).

**FIGURE 4:**
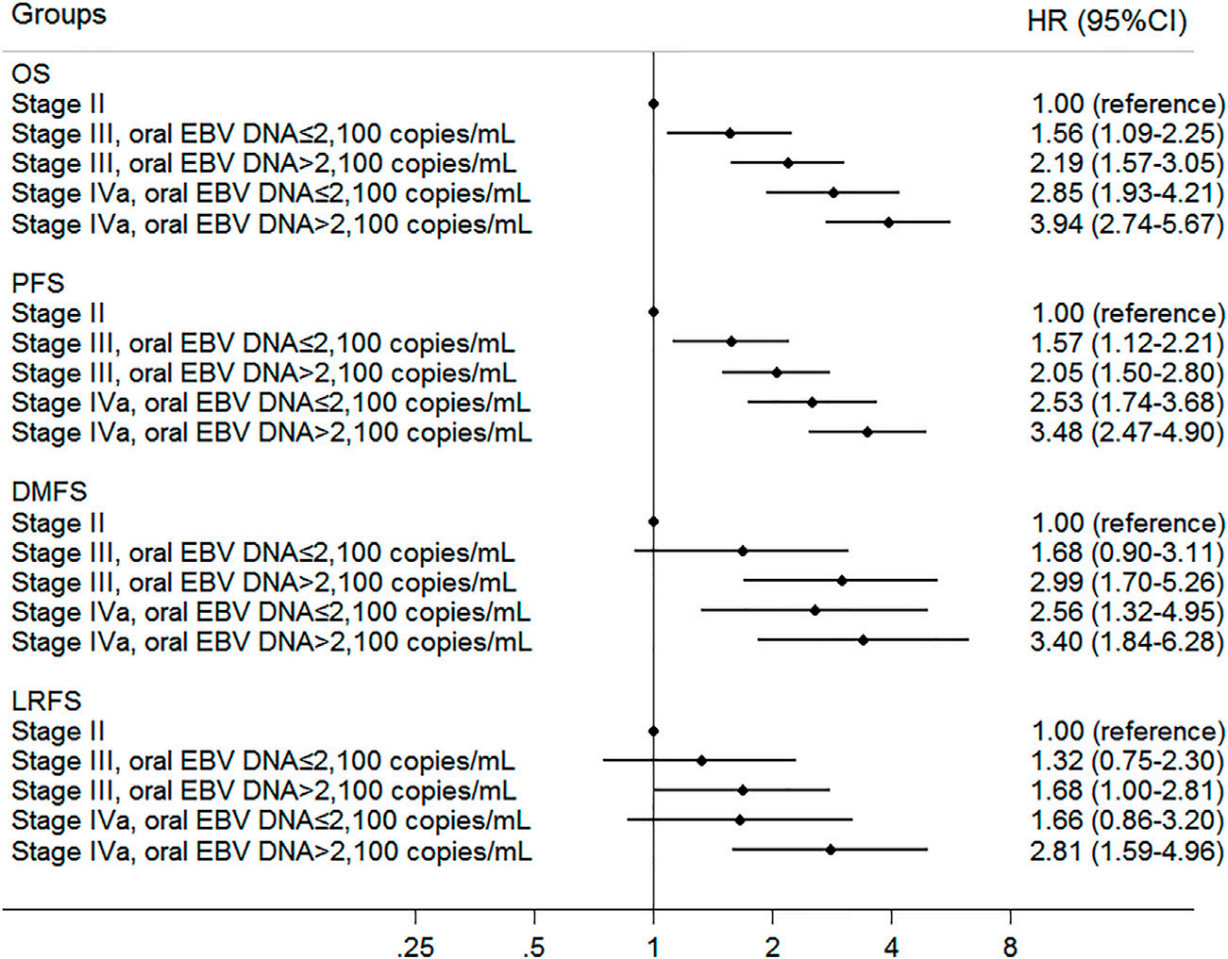
Multivariate associations of new groups defined by oral EBV DNA level and TNM staging with survivals in all 1,194 NPC patients. Survivals included overall survival (OS), progression-free survival (PFS), distant metastasis–free survival (DMFS), and locoregional relapse-free survival (LRFS).

To refine the risk stratification for NPC patients, all patients were subdivided into more subgroups based on TNM staging combined with an oral EBV DNA level. Specifically, patients were grouped into five new groups: stage II, low EBV in stage III, high EBV in stage III, low EBV in stage IVa, and high EBV in stage IVa. Survival curves suggested that the new grouping provided more detailed risk discrimination. With the increase of the clinical stage and oral EBV DNA levels, the hazard of death and progression presented a gradually increasing trend (Figure 3). Interestingly, we observed that patients in stage IVa with the low oral EBV DNA load had similar DMFS and LRFS compared to patients in stage III with the high oral EBV DNA load (Figures 3C,D). Compared to TNM staging, the new grouping combining the oral EBV DNA load and the TNM stage showed improved risk discrimination [concordance index (c-index), 0.636 vs. 0.617 for OS, 0.621 vs. 0.603 for PFS]. Multivariate Cox analyses further confirmed these observations (Figure 4). Furthermore, similar trends were observed as the whole set during subgroup analyses in pretreatment and mid-treatment subsets of patients (Supplementary Figure 2 and Supplementary Figure 3).

### Correlation Between the Oral EBV DNA Load and the Plasma EBV DNA Load

In a subset of 229 NPC patients with available data on the plasma EBV load, we found no significant correlation between the oral EBV load and the plasma EBV load (Spearman *R* ^2^ = 0.062, *p* = 0.349, Supplementary Figure 4).

## Discussion

Saliva has become an attractive hotspot in the identification of cancer biomarkers (Bonne and Wong, 2012) because of simple and non-invasive sample collection, which dramatically reduces the discomfort and incompliance of patients with repeated sampling for disease monitoring. The use of saliva as a test specimen is also a cost-efficient method without the need for highly trained professionals, and the processing of saliva is much easier and less demanding than blood, requiring no in-time centrifugation. The convenience and flexibility of the saliva sample collection make “self-sampling” feasible for disease screening and surveillance in the future, which provides more potential application scenarios. Recently, researchers have reported salivary high-risk human papillomavirus (HPV) DNA could serve as a biomarker for the detection and prognostic monitoring of oropharyngeal cancer (Weeramange et al., 2021). Oral cavity is an important place of EBV infection and existence, where oral epithelium infected with EBV routinely secretes viral particles into saliva (Ling et al., 2003a), which is primarily responsible for the human-to-human transmission of EBV. The salivary EBV load has been explored as biomarkers in various diseases including lymphoproliferative disorders (Saito et al., 1991), periodontitis (Dawson et al., 2009), and human immunodeficiency virus (HIV) (Ling et al., 2003b), but rarely in patients with NPC (Pow et al., 2011; Xue et al., 2018) or other EBV-related malignancies.

A previous study with 46 NPC patients reported that patients with T3–4 stages had the higher posttreatment salivary EBV DNA load (median 50,040 copies/mL) than patients with T1–2 stages (54 copies/mL) (Pow et al., 2011). Here, we also observed that NPC patients at the advanced T stage (T3–4) had significantly higher pretreatment (median 19,895 vs. 4,189 copies/mL) and mid-treatment (median 1,141 vs. 0 copies/mL) oral EBV DNA load than patients at the early T stage (T1–2). Additionally, the positive association between the T stage and EBV DNA levels in the oropharynx was consistent with that observed in plasma or nasopharynx brushings (Lo et al., 1999; Tong et al., 2002; Zheng et al., 2015; Zhang et al., 2018). EBV DNA in plasma or nasopharynx brushings of NPC patients are considered to be “naked” DNA fragments mainly released by apoptotic tumor cells, though, which remains to be defined (Chan et al., 2003; Stevens et al., 2005; Ramayanti et al., 2017). Compared to patients with the early T stage, more tumor cells releasing EBV are expected in advanced T stage NPC patients, which may partly explain why a higher EBV DNA level was obtained in blood or nasopharynx. Given the proximity of the nasopharynx and oropharynx, we hypothesize that the respiratory and swallowing movements would result in the transfer of viruses from the tumor site in the nasopharynx to the oropharynx, which affects the number of EBV in the oropharynx. This may partly explain why higher oral EBV DNA levels were obtained in advanced T stage patients and provided a reasonable explanation that patients with advanced NPC tended to have higher oral EBV DNA levels and presented poorer outcomes, albeit more detailed mechanisms remain to be further studied.

In this study, the EBV DNA load in mid-treatment mouthwashes was found significantly lower than that in the pretreatment samples. This finding agreed with the trends observed in plasma that the EBV DNA load decreased with the course of treatment (Huang et al., 2019; Lv et al., 2019). This is easy to understand. After treatment, the tumor burden decreases, and so does the patient’s EBV DNA load, further supporting the association between the oral EBV DNA load and tumor burden. However, our result disagreed with that of Pow et al. (2011) who reported that the median posttreatment salivary EBV DNA load (3,007 copies/mL) was higher than the pretreatment level (99 copies/mL) in 46 NPC patients. This discrepancy could be due to various reasons. First, the sample size of that study was small, and the results were likely to be highly biased. Second, the two studies had inconsistent definitions of pre- and post-treatment, with pre- and post-treatment in that study referring to before and 2 months after radiotherapy, whereas in our study, it was before and after the initiation of any treatment (induction chemotherapy/radiotherapy/chemoradiotherapy), respectively. Third, Lv et al. (2019) reported inter-patient heterogeneity in plasma EBV DNA response to treatment, with the virus in some patients being completely cleared, some persisting, and some rebounding. So, it is possible that patients in our study and Pow’s responded differently to treatment, leading to different directions of change in the oral EBV DNA load before and after treatment. Anyway, the dynamic changes of the oral EBV DNA load throughout treatment need to be studied further by sequential monitoring of individuals with a sufficient sample size. Nevertheless, regardless of how it changes with treatment course, our study showed that the high oral EBV DNA load was a prognostic risk factor for LA-NPC patients, both pre- and mid-treatment. This result was consistent with previous findings regarding another well-proven prognostic biomarker, plasma EBV DNA (Lin et al., 2004; Leung et al., 2014; Liu et al., 2015; Huang et al., 2019).

With the relatively large cohort and long-term follow-ups, here we demonstrated that the oral EBV DNA load was an independent indicator for NPC survivals, mainly OS, PFS, and DMFS, while its associations with LRFS were not that significant. Since the HRs of the associations between the oral EBV DNA load and LRFS have been always greater than 1, we consider the statistical insignificance in some analyses may be due to the small number of locoregional recurrence events followed up (179 events) in our study. Furthermore, we found that the oral EBV DNA load was able to complement TNM staging in the prognosis prediction of NPC, which may be helpful in treatment guidelines for the best benefit of NPC patients. Unlike circulating cell-free EBV DNA that has been studied for decades of years and is considered to be released mainly from the tumor cells (Mutirangura et al., 1998), little is known about the situation of oral EBV in NPC or other EBV-associated cancers. Our preliminary analysis in a limited subset of 229 patients with available pretreatment plasma EBV DNA data demonstrated no significant correlation between the oral and plasma EBV DNA loads, which may partly indicate the different origins of the two biomarkers. In addition to tumor cells, salivary EBV DNA of NPC patients may also originate from oropharyngeal epithelium and B lymphocytes (Hadinoto et al., 2009). Our previous preliminary study investigated the EBV presence in fractionating mouthwash samples of NPC patients by low- and ultra-speed centrifugation and found the EBV DNA was present in the separate component fractions of mouthwash, such as in the pellets from ultra-speed centrifugation, fragmented DNA from ultra-speed centrifugation, and pellets from low-speed centrifugation, which indicated the existence of cell-free EBV, fragmented EBV DNA, and cell-derived EBV, respectively (Xue et al., 2018). The oral cavity is such a complex environment that EBV reproduction in the oropharynx may be altered by various endogenous and exogenous factors, including but not limited to the local tumor, immune microenvironment, miR-200 family members (Lin et al., 2016), cigarette smoking, and other lifestyle factors (He et al., 2019). Further studies are warranted to investigate the origin, half-life, and molecular characteristics of oral EBV DNA, and to evaluate the relationships of EBV DNA load in the oropharynx with blood or nasopharynx. Our preliminary study on the prognostic value of oral EBV in NPC patients may be helpful to expand our understanding of EBV carcinogenesis and provide some clues for further in-depth mechanistic research in the future.

Since only one mouthwash sample was taken per patient in this study, we could not intuitively compare the oral EBV DNA load changes during treatment and progression, and assess the prognostic value of such changes. Hence, it would be of great significance to further clarify these issues in future well-designed dynamic studies, which could provide more insights into the oncogenic and the prognostic role of oral EBV DNA for NPC. It is worth mentioning that we have previously observed a decrease in the oral EBV DNA load among NPC patients compared to healthy controls (Xue et al., 2018). We think that the overall decrease of the oral EBV DNA load in NPC patients was possibly the result of tumorigenesis, during which the lytic replication of EBV in the oral cavity may be suppressed by some mechanisms that have not yet been well elucidated, such as the epigenetic modification of EBV genes or the dysregulation of the host’s regulatory genes. Hence, the associations between the oral EBV DNA load and NPC risk or NPC survival deserve further in-depth exploration.

In the efforts to promote the clinical application of salivary biomarkers, the discovery of effective salivary biomarkers, the understanding of their origin and biological mechanism, the optimization of biodetection methods, and the standardization of saliva sampling procedures based on the type of downstream assays are all critical. We will continue to make more explorations and discoveries in these aspects.

In summary, through this prospective observational clinical study, we first comprehensively evaluated the prognostic value of the oral EBV DNA load in LA-NPC patients and demonstrated that a high level of the oral EBV DNA load was an independent predictor of poor prognosis for LA-NPC patients, which could be a feasible consideration to complement TNM staging for NPC risk stratification.

## Data Availability

The original contributions presented in the study are included in the article/Supplementary Material; further inquiries can be directed to the corresponding author.
