# Supplementary material for: Prognostic Value of Oral Epstein–Barr Virus DNA Load in Locoregionally Advanced Nasopharyngeal Carcinoma
Source: Front Mol Biosci. 2022 Jan 13;8:757644. doi: 10.3389/fmolb.2021.757644 (PMC8793774; doi:10.3389/fmolb.2021.757644)
Supplement: Supplementary file 1 [file DataSheet1.docx]

**Supplementary Methods**

***Oral samples collection, DNA extraction and EBV DNA load quantification***

Oral EBV DNA load was measured in the collected mouthwashes before the commencement of any therapy (pretreatment), or between the start of treatment and the end of radiotherapy/chemoradiotherapy (mid-treatment). All mouthwash samples were collected and processed by standard procedures. Each patient was instructed to rinse their mouth with 10 mL physiological saline (0.9% NaCl) for 30s without eating or drinking 30 min before sampling. Mouthwash DNA was isolated using a phenol-chloroform procedure. Each 10 mL mouthwash sample was added with 500 μL of 10% SDS and 7 μL of 20 mg/mL RNase and incubated for 30 min at 37°C. After addition of 20 μL of 20 mg/mL proteinase K, the samples were incubated at 50°C for 30 min. Next, an equal volume of phenol: chloroform: isopentanol (25:24:1, pH 8.0) was added and then the mixture was centrifuged at room temperature for 10 min at 5000 xg. The supernatant was carefully taken and extracted again with equal volume of chloroform, followed by addition of 1/10 volume of 3M NaAc (pH 5.0) and double volume of ice-cold 100% ethanol. After cooling at -20°C for 20 min, the precipitated DNA was pelleted and washed twice with 70% ethanol and finally dissolved in 50 μL nuclease-free water.

The EBV DNA load was quantified using real-time quantitative PCR assay targeting a 76 bp segment in the BamHI-W region of the EBV genome. The primers and probes were synthesized by Thermo Fisher Scientific (MA, USA) and the sequences were as follows: forward primer, 5-CCCAACACTCCACCACACC-3; reverse primer, 5-TCTTAGGAGCTGTCCGAGGG-3; TaqMan probe, 5-FAM- CACACACTACACACACCCACCCGTCTC-TAMRA-3. The qPCR reaction mixture consisted of 1x LightCycler Master Mix for Hybridization Probes (Roche), 500 nM each PCR primer, 100 nM TaqMan probe , and 2 μL DNA template in a final reaction volume of 8 μL. PCR reaction was performed in LightCycler 480 under the following conditions: 5 min at 95 °C, followed by 45 cycles of 30s at 95 °C, 30s at 60°C and 15s at 72°C, and a final cooling step at 40°C for 30 s. Plasmid DNA containing BamHI-W region of the EBV genome was used as positive control and serially diluted as standards for absolute quantification (10^2^, 10^3^, 10^4^, 10^5^, 10^6^ and 10^7^ copies per 2 μL). Nuclease- free water was included as a negative control. To better measure the reliability and reproducibility of the EBV DNA test, all testing samples and quantification standard samples were tested in duplicate. Oral EBV DNA load was expressed as the copies of EBV-DNA per mL of mouthwash.

**Supplementary Table 1** Univariate associations of clinical characteristics with oral EBV DNA load at different treatment time-points in NPC patients

| Variables | **Pre-treatment ^a^**  **n=636** | |  | **Mid-treatment ^a^**  **n=558** | |  | **Overall ^a^**  **n=1,194** | |
| --- | --- | --- | --- | --- | --- | --- | --- | --- |
|  | OR (95%CI) | *P* |  | OR (95%CI) | *P* |  | OR (95%CI)^b^ | *P* |
| **Age** (≥45 vs.<45.) | 1.34 (0.97-1.86) | 0.074 |  | 1.68 (1.20-2.36) | 0.002 |  | 1.50 (1.20-1.89) | <0.001 |
| **Sex** (Male vs. Female) | 1.69 (1.19-2.41) | 0.003 |  | 1.79 (1.21-2.65) | 0.003 |  | 1.66 (1.29-2.15) | <0.001 |
| **Smoking status** (Yes vs. No) | 1.28 (0.92-1.76) | 0.143 |  | 1.53 (1.09-2.14) | 0.014 |  | 1.36 (1.08-1.71) | 0.008 |
| **Tumor stage** (T3-4 vs. T1-2) | 1.66 (1.17-2.34) | 0.004 |  | 1.76 (1.17-2.62) | 0.006 |  | 1.55 (1.21-2.00) | 0.001 |
| **N stage** (N2-3 vs. N0-1) | 0.72 (0.51-1.02) | 0.062 |  | 0.66 (0.47-0.93) | 0.018 |  | 0.66 (0.52-0.83) | 0.001 |
| **Overall stage** (III vs. II) | 1.16 (0.77-1.75) | 0.469 |  | 1.16 (0.68-1.97) | 0.580 |  | 1.02 (0.75-1.40) | 0.885 |
| **Overall stage** (IVa vs. II) | 1.41 (0.88-2.26) | 0.154 |  | 1.32 (0.75-2.32) | 0.331 |  | 1.16 (0.82-1.64) | 0.406 |

^a^ Pretreatment, before any treatment; Mid-treatment, after the beginning of treatment but before the end of radiotherapy /chemoradiotherapy; Overall, all patients included.

**Supplementary Table 2.** Multivariate Cox regression analysis of oral EBV DNA load segregated by different cutoffs with NPC survivals

| EBV DNA ^a^ | OS | |  | PFS | |  | DMFS | |  | LRFS | |
| --- | --- | --- | --- | --- | --- | --- | --- | --- | --- | --- | --- |
|  | HR (95%CI) ^b^ | *P* |  | HR (95%CI) ^b^ | *P* |  | HR (95%CI) ^b^ | *P* |  | HR (95%CI) ^b^ | *P* |
| **Overall ^c^** |  |  |  |  |  |  |  |  |  |  |  |
| >2,100 vs. ≤2,100 copy/mL ^d^ | 1.45 (1.20-1.74) | <0.001 |  | 1.38 (1.16-1.65) | <0.001 |  | 1.66 (1.25-2.21) | 0.001 |  | 1.43 (1.05-1.96) | 0.023 |
| >4,450 vs. ≤4,450 copy/mL ^f^ | 1.28 (1.07-1.53) | 0.007 |  | 1.23 (1.04-1.46) | 0.017 |  | 1.35 (1.03-1.77) | 0.032 |  | 1.37 (1.01-1.86) | 0.040 |
| **Pretreatment ^c^** |  |  |  |  |  |  |  |  |  |  |  |
| >2,100 vs. ≤2,100 copy/mL ^de^ | 1.51 (1.16-1.98) | 0.002 |  | 1.43 (1.11-1.84) | 0.006 |  | 1.60 (1.05-2.45) | 0.030 |  | 1.22 (0.79-1.89) | 0.377 |
| >13,000 vs. ≤13,000 copy/mL^f^ | 1.34 (1.05-1.72) | 0.019 |  | 1.30 (1.03-1.64) | 0.030 |  | 1.29 (0.88-1.89) | 0.199 |  | 1.24 (0.82-1.87) | 0.310 |
| **Mid-treatment ^c^** |  |  |  |  |  |  |  |  |  |  |  |
| >2,100 vs. ≤2,100 copy/mL ^d^ | 1.35 (1.03-1.76) | 0.030 |  | 1.31 (1.02-1.70) | 0.037 |  | 1.77 (1.18-2.64) | 0.006 |  | 1.66 (1.05-2.62) | 0.030 |
| >230 vs. ≤230 copy/mL ^e^ | 1.22 (0.94-1.60) | 0.140 |  | 1.23 (0.95-1.59) | 0.114 |  | 1.68 (1.12-2.53) | 0.013 |  | 1.48 (0.94-2.35) | 0.093 |
| >400 vs. ≤400copy/mL ^f^ | 1.25 (0.95-1.63) | 0.109 |  | 1.25 (0.97-1.62) | 0.086 |  | 1.71 (1.14-2.57) | 0.010 |  | 1.46 (0.92-2.31) | 0.107 |

^a^ EBV DNA load in mouthwashes

^b^ Adjusted by age (≥45 vs. <45), sex, T stage, N stage, smoking status (yes or no), IC (yes or no), CCRT (yes or no), and radiotherapy technology.

^c^ Overall, all patients included; Pretreatment, before any treatment; Mid-treatment, between the start of treatment and the end of radiotherapy/chemoradiotherapy.

^d^ determined by the receiver operating characteristic (ROC) curve analysis for OS in all population

^e^ determined by the receiver operating characteristic (ROC) curve analysis for OS in corresponding subgroup

^f^ determined by the median value in the corresponding subgroup

Abbreviations: EBV= Epstein-Barr virus, NPC= nasopharyngeal carcinoma, OS= overall survival, PFS= progression-free survival, DMFS= distant metastasis-free survival, LRFS=locoregional relapse-free survival, IC= induction chemotherapy, CCRT=concurrent chemoradiotherapy.

**Supplementary Table 3.** Multivariate Cox regression analysis of oral EBV DNA load by different gradings with survivals in 1,194 NPC patients

| EBV DNA ^a^ | n | OS | |  | PFS | |  | DMFS | |  | LRFS | |
| --- | --- | --- | --- | --- | --- | --- | --- | --- | --- | --- | --- | --- |
|  |  | HR (95%CI) ^b^ | *P* |  | HR (95%CI) ^b^ | *P* |  | HR (95%CI) ^b^ | *P* |  | HR (95%CI) ^b^ | *P* |
| **Tripartite categories** ^c^ |  |  |  |  |  |  |  |  |  |  |  |  |
| ≤2100 | 535 | Reference |  |  | Reference |  |  | Reference |  |  | Reference |  |
| 2100~53800 | 329 | 1.41 (1.14-1.75) | 0.002 |  | 1.33 (1.09-1.64) | 0.006 |  | 1.64 (1.18-2.28) | 0.003 |  | 1.18 (0.81-1.72) | 0.395 |
| >53800 | 330 | 1.48 (1.20-1.84) | <0.001 |  | 1.44 (1.17-1.77) | 0.001 |  | 1.68 (1.20-2.34) | 0.002 |  | 1.72 (1.21-2.44) | 0.003 |
| *P-*trend |  |  | <0.001 |  |  | <0.001 |  |  | 0.001 |  |  | 0.003 |
| **Tripartite categories** ^d^ |  |  |  |  |  |  |  |  |  |  |  |  |
| 0 | 453 | Reference |  |  | Reference |  |  | Reference |  |  | Reference |  |
| 0~41305 | 370 | 1.29 (1.04-1.60) | 0.021 |  | 1.23 (1.00-1.52) | 0.048 |  | 1.49 (1.07-2.09) | 0.019 |  | 1.11 (0.75-1.62) | 0.606 |
| >41305 | 371 | 1.41 (1.14-1.76) | 0.002 |  | 1.37 (1.11-1.69) | 0.003 |  | 1.60 (1.14-2.24) | 0.006 |  | 1.61 (1.13-2.30) | 0.009 |
| *P-*trend |  |  | 0.002 |  |  | 0.003 |  |  | 0.006 |  |  | 0.009 |
| **Quadripartite categories** ^e^ |  |  |  |  |  |  |  |  |  |  |  |  |
| ≤2100 | 535 | Reference |  |  | Reference |  |  | Reference |  |  | Reference |  |
| 2100-23900 | 220 | 1.51 (1.19-1.92) | 0.001 |  | 1.40 (1.12-1.77) | 0.004 |  | 1.81 (1.27-2.60) | 0.001 |  | 1.20 (0.78-1.84) | 0.402 |
| 23900-132300 | 219 | 1.21 (0.94-1.54) | 0.133 |  | 1.23 (0.97-1.54) | 0.084 |  | 1.21 (0.82-1.78) | 0.338 |  | 1.33 (0.89-2.00) | 0.169 |
| >132300 | 220 | 1.71 (1.34-2.18) | <0.001 |  | 1.57 (1.24-1.99) | <0.001 |  | 2.11 (1.46-3.05) | <0.001 |  | 1.84 (1.24-2.73) | 0.002 |
| *P-*trend |  |  | <0.001 |  |  | <0.001 |  |  | 0.001 |  |  | 0.003 |
| **Quadripartite categories** ^f^ |  |  |  |  |  |  |  |  |  |  |  |  |
| 0 | 453 | Reference |  |  | Reference |  |  | Reference |  |  | Reference |  |
| 0-14130 | 247 | 1.28 (1.00-1.63) | 0.049 |  | 1.21 (0.96-1.53) | 0.102 |  | 1.52 (1.05-2.21) | 0.027 |  | 1.02 (0.65-1.58) | 0.945 |
| 14130-106000 | 247 | 1.20 (0.94-1.53) | 0.141 |  | 1.20 (0.96-1.52) | 0.115 |  | 1.26 (0.86-1.84) | 0.241 |  | 1.34 (0.90-2.00) | 0.151 |
| >106000 | 247 | 1.63 (1.28-2.08) | <0.001 |  | 1.52 (1.20-1.91) | <0.001 |  | 1.95 (1.35-2.82) | <0.001 |  | 1.73 (1.17-2.57) | 0.007 |
| *P-*trend |  |  | <0.001 |  |  | 0.001 |  |  | 0.002 |  |  | 0.005 |

^a^ EBV DNA load in mouthwashes

^b^ Adjusted by age (≥45 vs. <45), sex, T stage, N stage, smoking status (yes or no), IC (yes or no), CCRT (yes or no), and radiotherapy technology.

^c^ cutoff values defined by the unified cutoff 2100 and the median for those with oral EBV DNA above 2,100 copy/ml

^d^ cutoff values defined by 0 and the median for those with detectable oral EBV DNA

^e^ cutoff values defined by the unified cutoff 2100 and the tertiles for those with oral EBV DNA above 2,100 copy/ml

^f^ cutoff values defined by 0 and the tertiles for those with detectable oral EBV DNA

Abbreviations: EBV= Epstein-Barr virus, NPC= nasopharyngeal carcinoma, OS= overall survival, PFS= progression-free survival, DMFS= distant metastasis-free survival, LRFS=locoregional relapse-free survival, IC= induction chemotherapy, CCRT=concurrent chemoradiotherapy.


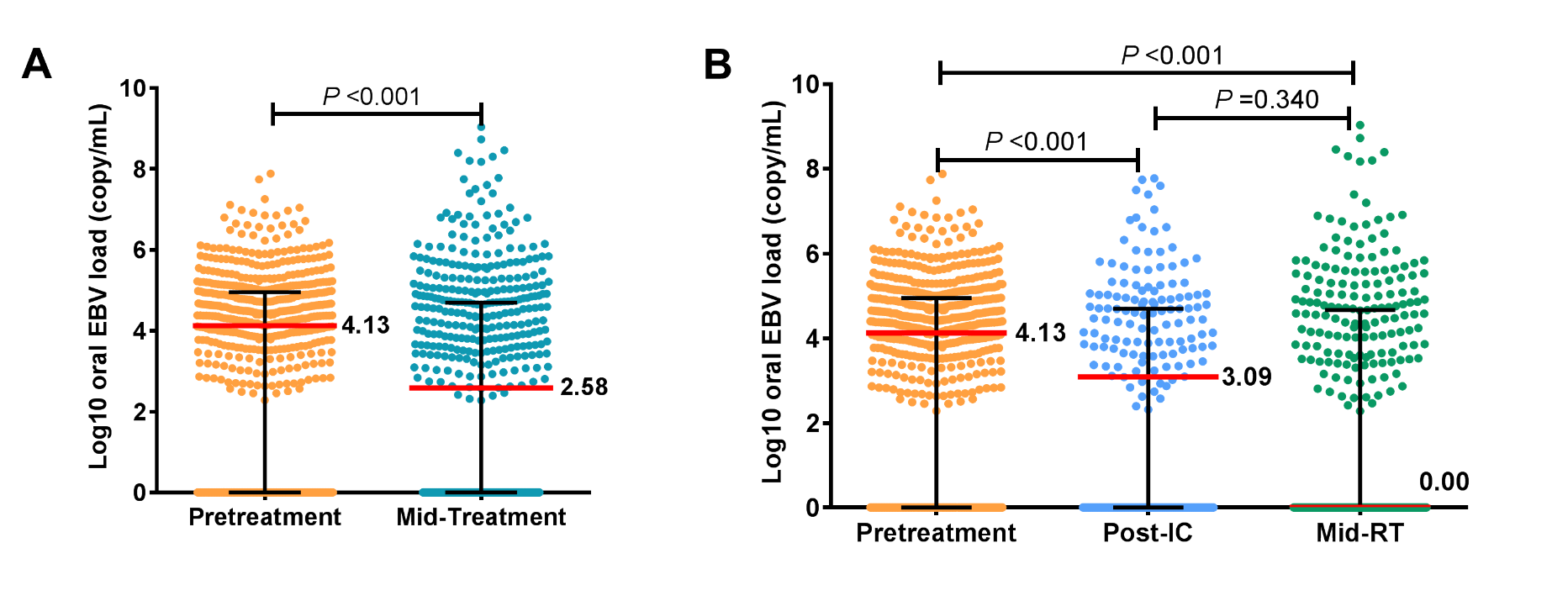


**Supplementary Figure 1. Oral EBV DNA load of patients with locoregional advanced NPC by different treatment stages.** (A) Patients were grouped into 2 stages of treatment, Pretreatment (sampling before any treatment) and Mid-treatment (sampling between the start of treatment and the end of radiotherapy/chemoradiotherapy. (B) Patients were grouped into 3 stages of treatment, Pretreatment, Post-IC (sampling between the start of induction chemotherapy and the start of radiotherapy/chemoradiotherapy), and Mid-RT (sampling during radiotherapy/chemoradiotherapy).


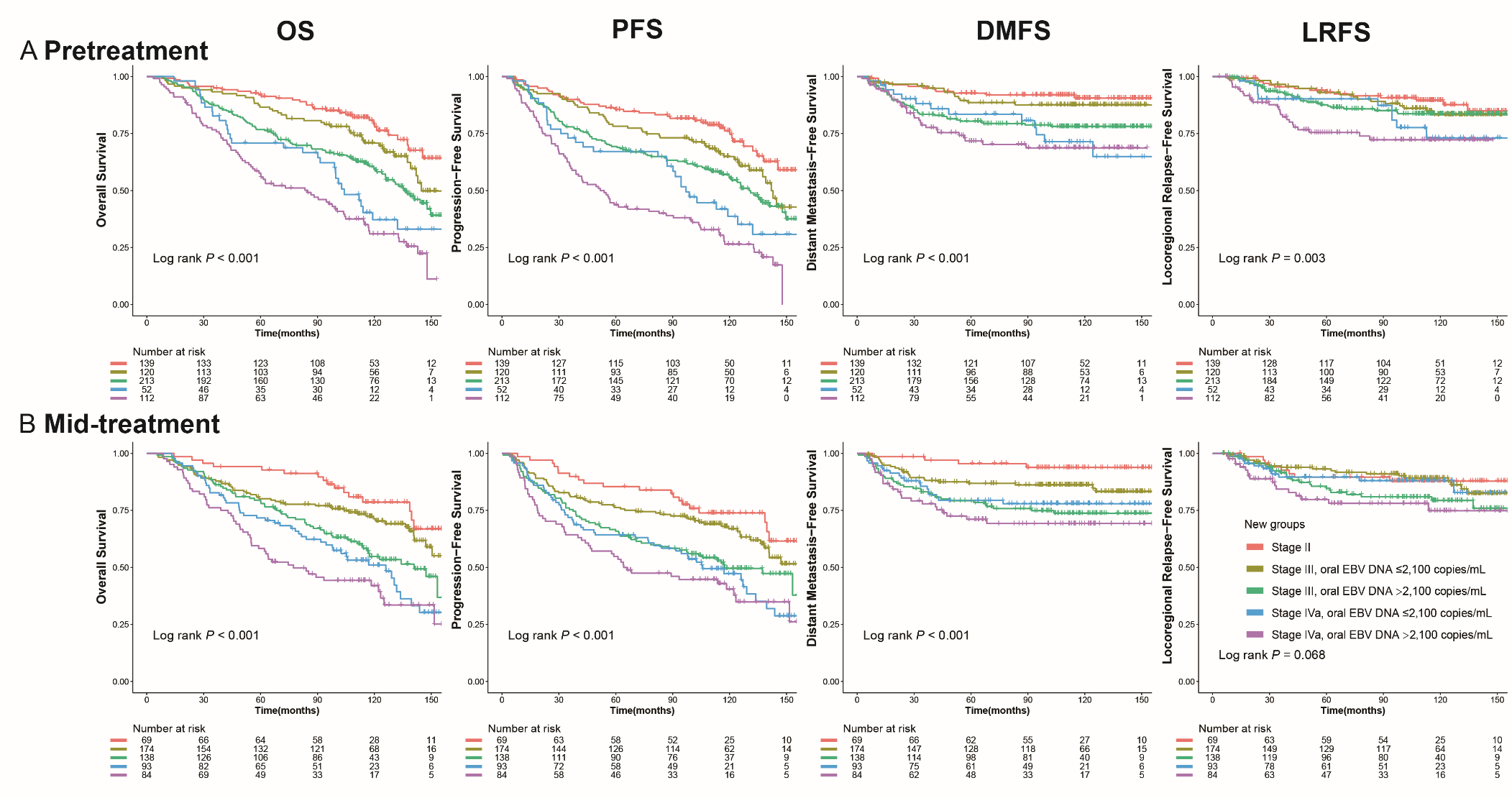


**Supplementary Figure 2. Kaplan-Meier survival curves for NPC survivals segregated by oral EBV DNA levels and TNM staging**: (A) pretreatment; (B) mid-treatment. OS=overall survival, PFS= progression-free survival, DMFS=distant metastasis-free survival, LRFS=locoregional relapse-free survival.


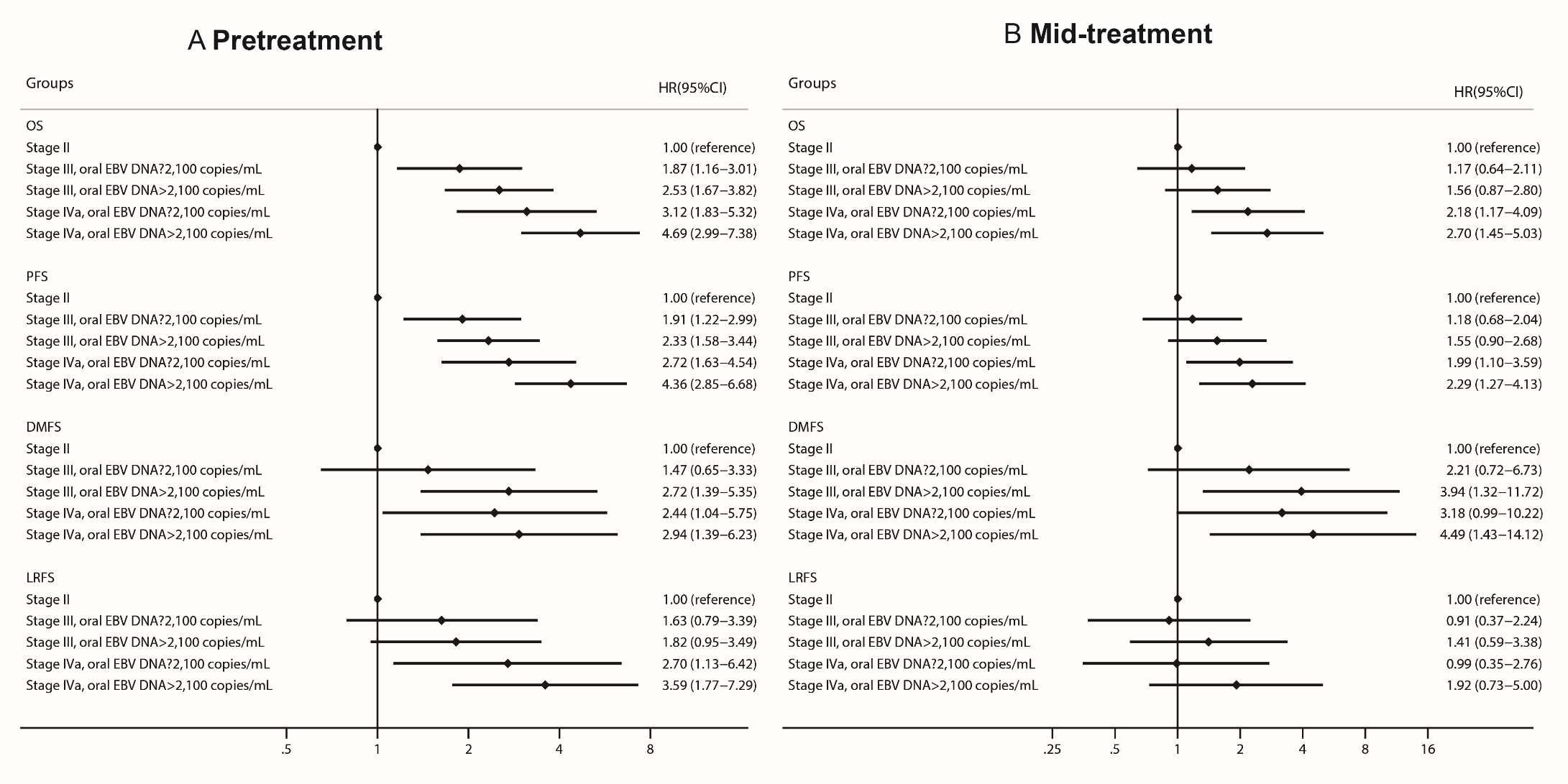


**Supplementary Figure 3. Multivariate associations of new groups defined by oral EBV DNA load and TNM staging with NPC survivals in patients at different treatment stages:** (A) Pretreatment; (B) Mid-treatment.


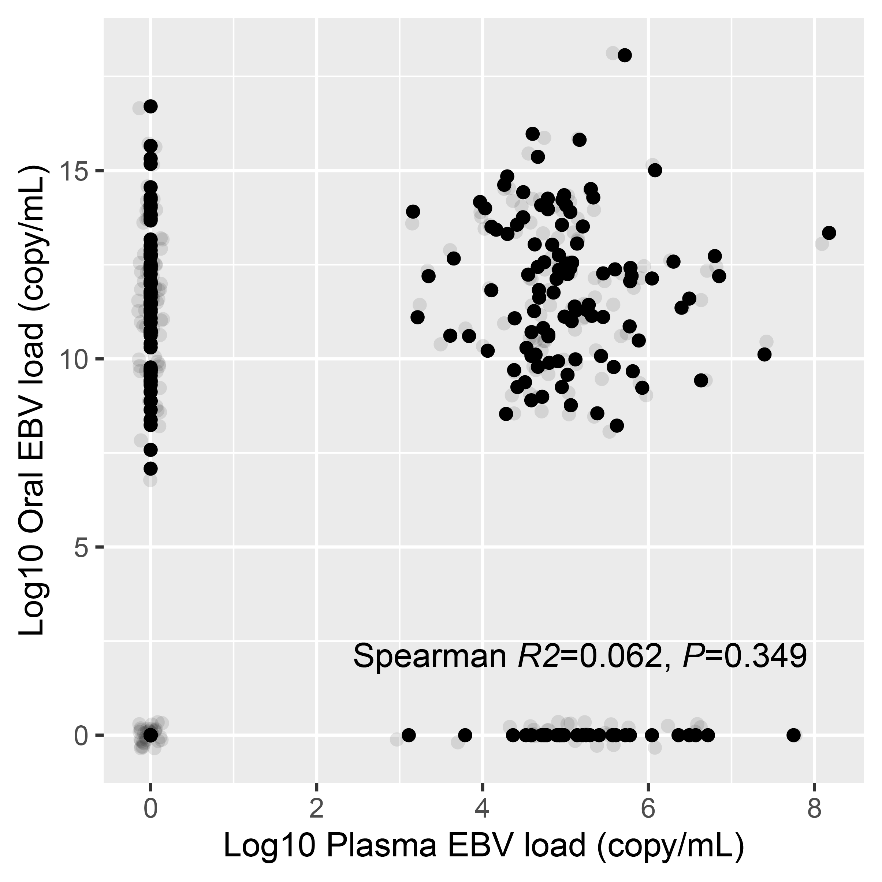


**Supplementary Figure 4. The relationship between oral EBV DNA load and plasma EBV DNA load in 229 NPC patients.**
